# The Cue-Approach Task as a General Mechanism for Long-Term Non-Reinforced Behavioral Change

**DOI:** 10.1038/s41598-018-21774-3

**Published:** 2018-02-26

**Authors:** Tom Salomon, Rotem Botvinik-Nezer, Tony Gutentag, Rani Gera, Roni Iwanir, Maya Tamir, Tom Schonberg

**Affiliations:** 1 Faculty of Life Sciences, Department of Neurobiology, Tel Aviv University, Tel Aviv, Israel; 2 Sagol School of Neuroscience, Tel Aviv University, Tel Aviv, Israel; 3 Department of Psychology, The Hebrew University of Jerusalem, Jerusalem, Israel

## Abstract

Recent findings show that preferences for food items can be modified without external reinforcements using the cue-approach task. In the task, the mere association of food item images with a neutral auditory cue and a speeded button press, resulted in enhanced preferences for the associated stimuli. In a series of 10 independent samples with a total of 255 participants, we show for the first time that using this non-reinforced method we can enhance preferences for faces, fractals and affective images, as well as snack foods, using auditory, visual and even aversive cues. This change was highly durable in follow-up sessions performed one to six months after training. Preferences were successfully enhanced for all conditions, except for negative valence items. These findings promote our understanding of non-reinforced change, suggest a boundary condition for the effect and lay the foundation for development of novel applications.

## Introduction

Behavioral change is an essential tool to improve health and quality of life, from treating addictions to eating and mood disorders^1–3^. Scientific research on behavioral change mainly focused on the effects of external reinforcements^2,4–7^ or altering the presentation of the decision problem^8–10^. Recently, the cue-approach training (CAT)^11^ paradigm was introduced as a successful method for enhancing preferences for food items, without external reinforcement, context change or self-control. Here, we test multiple hypotheses that are aimed to shed light on this mechanism by studying its generalizability to multiple stimuli and cues, as well as the long-term durability of the effect.

In the cue-approach task, participants initially indicated their preferences for a set of snack-food items by specifying their willingness to pay for each item in an auction procedure. Then, in the CAT, some of the items were consistently associated with a neutral auditory cue and a speeded button press response (these stimuli were termed ‘Go items’), whereas other stimuli were presented without a cue (‘No-Go items’). In the following probe phase, participants were asked to choose a snack they would like to eat at the end of the experiment. Each probe-choice comprised of a pair of items with similar initial values, in which only one of the two snacks was a Go item. Results showed that the mere association of snack-food images with a neutral auditory cue and a speeded button press, resulted in enhanced preferences for Go items over No-Go items. This preference change effect varied across different value categories – resulting in enhanced preferences for snack-food items of initial high-value, yet significantly less prominent change in preferences for low-value items. The effect was maintained two-months following training^11^. Additional studies with the cue-approach task^12^ found that for the behavioral change to take place, CAT required the presence of both a speeded button press response and a cue; i.e. CAT had no effect when training was conducted with an early cue onset which was followed with a full one second to respond. In addition, when training included only button press responses with no cues, enhanced preferences for Go items were not observed^12^. It was also found that CAT induces preference changes beyond the hand motor circuit itself, relying on a study that trained participants with the button press, whereas choices during probe were made using the eye gaze^12^. Veling *et al*.^13^ extended the range of consumable items for non-reinforced change to fruits and vegetables, and also found that the effect requires a time-restricted response. Finally, it was also shown that using CAT, it is possible to increase the odds of participants choosing low-value snack-food items over high-value items, in comparison to a baseline rate of choices between two non-cued items^14^.

Based on these findings, summarizing over 15 samples, the cue-approach task has been established as a replicable paradigm to induce preference changes for consumable food items, without external reinforcement. The underlying mechanism of this preference change has not yet been fully elucidated. Imaging studies showed a neural signature indicative of value change for the Go items in the ventromedial prefrontal cortex during choices^11,15^. Further imaging classification analyses were not able to clearly define its underlying basic cognitive constructs, but did show the involvement of frontal control networks following training^15^. Currently it is suggested that the underlying mechanism relies on attention^11,12,15^, inspired by its conceptual resemblance to the attentional boost effect^16^, where memory for task-irrelevant stimuli was enhanced when stimuli co-appeared with an irrelevant target cue. Thus far, the cue-approach effect, was demonstrated only on consumable items with a neutral auditory cue and up to a two-month follow up. The goal of the present study was to test multiple hypotheses in 10 independent samples using various non-consumable stimuli and different cues, as well as long-term follow-up sessions. This would promote our understanding of the underlying mechanism, as well as the boundary conditions of the cue-approach task as a general mechanism for long term non-externally reinforced change.

Our first research aim was to test whether CAT can enhance preferences for non-consumable items. To this end, we performed CAT with a variety of non-consumable stimuli, which have never been tested with CAT before. First, we tested the efficacy of CAT in changing preferences for face images (Experiment 1), which are important social stimuli and known to elicit preferences^17,18^. Second, we tested if CAT could be used to change preferences for more abstract stimuli, such as fractal art images (Experiment 2), which participants are unaccustomed to and are also more difficult to associate with semantic knowledge. Third, we continued to examine the importance of the stimuli’s affective valence. Research with external reinforcement procedures^19–21^ indicated an important interplay between the stimulus affective valence (aversive versus appetitive) and the required response (approach versus avoidance) – showing better association of approach responses with appetitive stimuli, and avoidance responses with aversive stimuli. To examine the importance of the stimuli’s valence, we performed two experiments using affective images from the International Affective Picture System^22^ (IAPS): one experiment was performed with positive IAPS stimuli (Experiment 3), and a second with negative IAPS stimuli (Experiment 4).

After examining the importance of different stimuli features (i.e., consumability, abstractness and affective valence), we sought to examine the importance of the cues’ nature. Previous studies suggested that cues of different modalities may alter performance in different tasks^23,24^. Since all of the previous experiments with CAT^11–15^ were conducted with a neutral auditory cue, we examined whether the cue’s auditory modality is a crucial feature of CAT. In Experiment 5 we tested whether training with a visual cue would also induce enhanced preferences for familiar snack-food items. In Experiment 6, we further tested the importance of the valence^19–21^ of the cue by associating appetitive snack-food items with an aversive tone cue. Here, we did not have a clear hypothesis if the aversive cue combined with the appetitive stimuli will lead to enhanced preferences. We put to the test two competing hypotheses – on the one hand, CAT with a neutral cue resulted in enhanced preferences for the associated items, and therefore if the valence of the cue is not a cardinal feature, we would expect enhanced preferences also with an aversive cue. On the other hand, the cue’s valence may be a fundamental factor, as association of stimuli with an aversive cue may result in reduced preferences via classical conditioning^25^.

To adhere to principles of replicability^26–28^ in Experiments 7–10 we performed improved replications of our novel findings with non-consumable items from Experiments 1–4. In all of the replications, we used a more extensive training protocol with both an auditory cue (Experiments 7–8) and a visual cue (Experiments 9–10). In Experiment 7 we replicated Experiment 1 with a new set of faces adapted from a more recent, better quality dataset. This was done following reports by participants that the stimuli used in Experiment 1 were outdated and not visually appealing. In Experiment 8 we replicated Experiment 2 with fractals. Finally, in Experiments 9–10 we replicated Experiments 3–4 with the positive and negative affective IAPS stimuli, with a visual cue during training. Replicating the results with a visual cue was important to ensure participants in the negative IAPS condition did not avert their gaze from the unpleasant images during training.

In addition to testing the generalizability of the effect with multiple non-consumable stimuli and cues, another central aim of the current study was to examine the long-term durability of non-reinforced behavioral change. Therefore, we invited participants from five experiments to an additional follow-up session. We assessed the long-term effect following a period of one-month (Experiment 7), two-months (Experiments 5 and 6), four-months (Experiment 2) and six-months (Experiment 8) after training. In these experiments, we employed the longest follow-up period to test the durability of CAT effect thus far. Demonstrating that the behavioral change effect is durable over a long period of time could point to its underlying mechanism that putatively effects the low-level representation of the items^29^, as well as potential applicability in inducing long-term change.

Based on previous findings^11–15^, we hypothesized that CAT would result in increased preferences for Go items (i.e. items that were previously associated with a cue and a response) over No-Go items with similar initial preferences. We also predicted that this behavioral change would be more robust for high-value items than for low-value items, as reported in most studies with CAT^11,12,15^, though not all of them^13^.

## Methods

### Participants

A total of 255 healthy participants participated in one of 10 independent experiments. In five experiments (Experiments 2 and 5–8), 84 participants agreed to return for an additional follow-up session (average of 67% retention rate of the first samples), one to six-months following their original participation date (for a demographic description of each experimental sample see Table 1). All participants had normal or corrected to normal vision and hearing, and gave their informed consent to participate in the experiments in return for monetary compensation or in return for course credit (course credit was granted to some of the participants in Experiments 3–4 and 9–10). The study was approved by the ethics committee of Tel Aviv University (Experiments 1–2 and 5–8) and by the ethics committee of the Hebrew University of Jerusalem (Experiments 3–4, and 9–10). All experiments were performed in accordance with relevant guidelines and regulations.

**Table 1:** Demographics.

Experiment Stimulus	Go cue	Sample Size (Excluded)	Females (Proportion)	Mean Age (*SD*)	Follow-up	
					Sample Size	Mean Interval^a^ (Range)
Exp. 1: Faces	Auditory	19 (12)	10 (53%)	24.42 (3.27)	—	—
Exp. 2: Fractals	Auditory	25 (3)	15 (60%)	23.04 (1.92)	14	115.6 (73–170)
Exp. 3: Positive IAPS	Auditory	27 (1)	17 (63%)	24.11 (2.62)	—	—
Exp. 4: Negative IAPS	Auditory	28 (5)	21 (75%)	25.04 (2.53)	—	—
Exp. 5: Snacks	Visual	25 (1)	16 (64%)	22.24 (2.30)	21	51.8 (34–69)
Exp. 6: Snacks	Auditory Aversive	25 (5)	15 (60%)	24.40 (2.15)	14	61.5 (31–105)
Exp. 7: Faces	Auditory	25 (1)	18 (72%)	25.16 (2.44)	18	33.2 (22–56)
Exp. 8: Fractals	Auditory	25 (2)	19 (76%)	24.20 (3.33)	16	183.9 (171–203)
Exp. 9: Positive IAPS	Visual	29 (3)	18 (62%)	23.27 (2.08)	—	—
Exp. 10: Negative IAPS	Visual	27 (3)	20 (74%)	23.67 (2.60)	—	—

^a^Mean interval in days from training to follow-up session.

Our target sample size of n = 25 for Experiments 2–10 was selected based on power analysis of 80% power to detect an effect with 0.05 significance. Power was calculated using a two-sided one-sample t-test, comparing the mean proportion of trials when high-value Go items were chosen, against a null hypothesis of 50% chance. Using a t-test provided both a good approximation of the logistic regression model results, and a straightforward analytical solution to perform the sample size power analysis. Data were obtained from previous experiments with CAT^11^ and Experiment 1 (power analysis was conducted using R’s pwr package^30^, and is available online at osf.io/h36vr). Final sample sizes ranged from 25–29; Data from participants beyond n = 25 were collected in order to guarantee the minimal n = 25 in case of exclusion following primary quality assurance of the data. All reported effects remain consistent when the participants beyond n = 25 were removed.

In addition to the reported 255 participants, across the 10 experiments 31 additional participants were excluded from final analyses (see Table 1 and Supplementary Table 1). Twenty-one participants were disqualified due to poor performance in training, an exclusion criteria adopted from previous cue-approach studies^11,12^; six participants due to technical problems with the apparatus running the experiment; two participants requested to quit; one participant entirely avoided choices of low-value snacks during the probe phase and one participant due to extreme intransitivity in initial preferences (transitivity score from initial preferences evaluation of 3.67 *SD* below the group mean).

## Materials

### Stimuli

Six different stimuli sets were used, each containing 60 identically-sized color images of either unfamiliar faces^31,32^ (two different datasets were used in Experiment 1 and Experiment 7), fractal art^33^ (Experiments 2 and 8), images from the International Affective Picture System (IAPS)^22^ of positive valence (Experiments 3 and 9) or negative valence (Experiments 4 and 10) and popular Israeli snack-food items^34^ (dataset created in our laboratory for Experiments 5 and 6).

**Figure 1:**
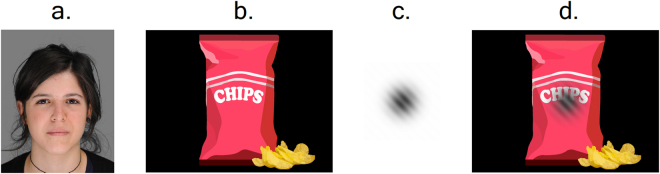
Stimuli and cue examples. (**a**) Example of a face stimulus with a neutral expression used in Experiment 7. (**b**) Illustration of a snack-food stimulus similar to the ones used in Experiments 5–6. (**c**) Semi-transparent Gabor used as a visual cue in Experiments 6 and 9–10. (**d**) Example of a snack-food stimulus with the visual cue overlaid on it. Images of faces are included with permission from the copyright holder^32^.

For Experiment 1 we used face images adapted from a functional MRI face localizer task^31^. The face stimuli included images of 30 male and 30 female front-facing individuals on a white background, sized 280 × 296 pixels. In Experiment 7, we performed an improved replication of the face experiment using a newer dataset^32^ with better quality face images. Stimuli included 30 male and 30 female front-facing figures, posing a neutral expression with limited facial hair and make-up. The original images were graphically edited using Adobe Photoshop, cropped to identical size (400 × 500 pixels) and the original green screen background was replaced with a homogenous gray background. In order to maintain similar position and relative proportion of the stimuli, faces were centered and aligned according to the location of the pupils (see example in Fig. 1a). Computer-generated fractal art stimuli^33^ used in Experiments 2 and 8 included 60 identical sized (576 × 432 pixels) color images.

For Experiments 3–4 and 9–10 with IAPS stimuli, images were selected based on the norms published by Lang *et al*.^22^. For the positive IAPS experiments (Experiments 3 and 9) we used 60 affective images, which were rated as inducing positive affect (valence *M* = 7.10 on a 1 to 9 scale, *SD* = 0.43; arousal *M* = 5.16 on a 1 to 9 scale, *SD* = 0.46). For the negative IAPS experiments (Experiments 4 and 10) we used 60 images rated as inducing negative affect (valence *M* = 2.87, *SD* = 0.55; arousal *M* = 5.50, *SD* = 0.78). Arousal ratings were similar in both datasets. In experiments with negative affective stimuli we also used three additional stimuli not from the official IAPS dataset, which were found to induce negative affect in another independent study^35^ (For a complete list of the IAPS stimuli and their codes, see Supplementary Table 2). All IAPS stimuli were rescaled to identical size of 533 × 400 pixels.

For Experiments 5 and 6, conducted with familiar snack-food items, we prepared a stimuli dataset with 60 local snacks^34^ (see Supplementary Table 3 for the full stimuli list). Snack-food images were of identical size (576 × 432 pixels) and presented on homogenous black background.

### Cues

We used three types of training cues: In Experiments 1–4, and 7–8, we used the original neutral auditory cue of a 180-ms sinusoidal wave tone, identical to the one used in previous cue-approach studies^11,12,15^; in Experiments 5, and 9–10, we used a visual cue of a 180-ms semi-transparent Gabor (see example in Fig. 1c,d); in Experiment 6 we used an aversive 300-ms auditory cue created with a cotangent function (provided as Supplementary Code).

The unpleasantness of the aversive cue was evaluated in a sub-sample of 18 out of 25 participants from Experiment 6. At the end of the experiment, the aversive cue was played, and participants were asked to rate its unpleasantness on a scale from 0 to 10 (with 10 being “Very unpleasant”). For these participants, we found that the sound was overall rated as unpleasant (median = 7, mean = 7.5, SD = 2.28).

### Stimuli presentation

Stimuli were presented using MATLAB (Mathworks, Inc. Natick, MA, USA), Psychtoolbox^36^ and Python-based Pygame^37^ packages, on 21.5 inch iMac or PC with a 21 inch screen. In order to induce the aversive cue in Experiment 6, the sound was played in a controlled high volume, using Plantronics BackBeat Pro noise-canceling headphones.

## Procedure

### Baseline evaluation of subjective preferences

Participants’ baseline subjective preferences for each of the stimuli in a given experiment were evaluated individually using two methods: an auction procedure in experiments with consumable snack-food items (Experiments 5 and 6), and a forced-choice binary ranking procedure for non-consumable items (Experiments 1–4 and 7–10). Based on these valuations, stimuli were rank-ordered individually for each participant, from the most liked item (rank 1) to the least liked item (rank 60).

#### Auction procedure for snack-food items

**Figure 2:**
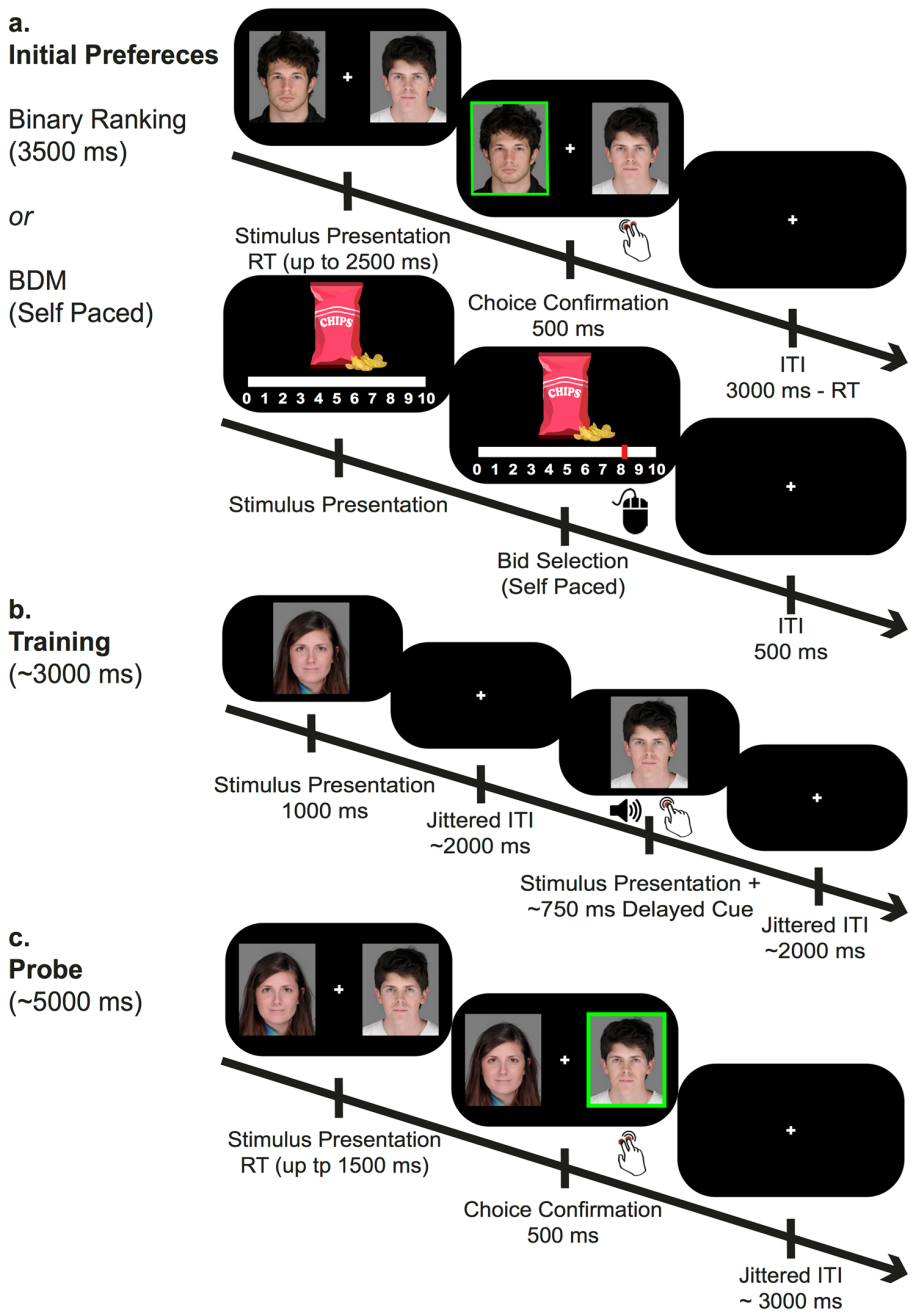
An example of the experimental procedure outline using face stimuli. Mean trial duration written in parenthesis, tilde sign indicates varying duration. (**a**) Initial preferences evaluation using either a binary ranking procedure (for non-consumable stimuli) or an auction procedure (for snack-food items, in Experiments 5–6). (**b**) In the training phase, Go items were consistently paired with a cue and a speeded button press. (**c**) In the probe phase participants chose their preferred stimulus between pairs of items with similar initial value, where only one was a Go item, previously associated with a cue during training. Images of faces are included with permission from the copyright holder^32^.

In experiments with snack-food items (Experiments 5 and 6), participants underwent an auction task based on the Becker DeGroot Marschak (BDM)^38^ auction procedure, to obtain participants’ willingness to pay, similarly to previous cue-approach studies^11–15^. Prior to their participation in the experiment, subjects were asked to fast for at least four hours. Upon arrival to the laboratory, participants received a sum of 10 New Israeli Shekels (~2.7$ US equivalent) to be used in the auction task. In this task, snack-food items were presented individually on the screen one at a time. Participants selected their bid for each item on a visual analogue scale using a mouse (see Fig. 2a). Participants were explicitly told that offering the amount they are willing to pay for each item was the best strategy for the task. Participants were informed in advance, that at the end of the experiment one trial will be chosen at random to be played out for real purchase from the experimenter. The computer randomly generated a counter bid, ranging from 0–10 with 0.5 increments. In case the participant’s bid was higher than the computer’s, he or she had won the bid and were required to buy the item in return for the computer bid price; otherwise, the participant could not purchase the item, but was left with the money designated for the auction at the beginning of the experiment.

### Binary ranking for non-consumables items

In experiments with non-consumable stimuli (i.e. faces, fractals and IAPS; Experiments 1–4 and 7–10), we used a forced-choice binary ranking procedure. In this task, 60 stimuli were randomly paired with each other to form 300 unique pairs. For each pair of stimuli, participants had 2500-ms to choose their preferred stimulus, followed by a 500-ms choice confirmation and 500-ms fixation cross (see Fig. 2a). In order to maintain balanced exposure, each stimulus was presented in exactly 10 pairs during the binary ranking phase. Participants did not receive any instructions to base their choices on any specific criteria (e.g. artistic value for fractals or attractiveness for faces).

Choices were then quantified into ranking scores. Based on the assumption of choice transitivity from Rational Choice Theory^39^, we used the outcomes from the set of binary choices in order to deduce individual preferences for the presented set of stimuli. That is, if stimulus A is preferred over B and stimulus B is preferred over C, then their respective ranks follow A≻B≻C. In order to maximize ranking validity and specificity we used the Colley Matrix algorithm^40^, designed to solve ranking problems with a relatively small number of binary outcomes. This binary ranking procedure resulted in a ranking list of the 60 stimuli, based on each participant’s individual preferences.

Colley Matrix ranking scores typically range from 0 (least liked) to 1 (most liked), with a fixed mean of 0.5. An intransitive choice pattern is characterized by densely distributed scores around the center of 0.5, while a distinct preferences pattern leads to more distributed ranking scores. From these rankings, we quantified a transitivity score for each participant as the standard deviation of the participant’s ranking scores. Participants who demonstrated extreme intransitive choice patterns (3 *SD* below the group mean), were excluded from final analyses.

### Cue-approach training

Following the baseline evaluation of subjective preferences, participants underwent a 40-minutes long CAT procedure, during which a consistent association was formed between some of the stimuli and a Go cue (see Fig. 2b). Each stimulus in the training set was presented individually on the screen for 1000-ms, once during each training run. Stimuli were randomly ordered and followed by a jittered fixation cross with an average duration of 2000-ms (*SD* = 1243-ms; range of 1000–6000-ms, 1000-ms intervals).

We used two formats of training designs, both were 40-mintues long, but the number of training runs (i.e. total number of presentations of each stimulus) was different, as well as the number of cued Go items. In the first, shorter-training design, used in Experiments 1–4 the training set consisted of all 60 stimuli (as in the original CAT study^11^), presented in 12 training runs. In the second design, used in Experiments 5–10, we extended the training phase, to test if an extensive training design would induce a stronger behavioral effect over the shorter one. In the more extensive design, the number of training runs was increased to 20 and the duration of each run was reduced by presenting only a subset of 40 stimuli, consisting of 20 high-value (ranked 3–22, above the median rank) and 20 low-value items (ranked 39–58).

One third of the training set items (20 out of 60 items in the shorter-training design) or 30% of items (12 out of 40 items in the extensive-training design) were associated with the Go cue. Participants were instructed to respond to the Go cue by pressing a keyboard button as fast as possible, before stimulus offset. Participants were not informed in advance that the association of stimuli with the cue would be consistent or which items would be Go items.

Items were assigned to be associated with the Go cue based on the previous baseline preferences evaluation task. Two sets of high-value stimuli with identical mean ranks and two sets of low-value stimuli with identical mean ranks were predetermined (see Supplementary Figure 1). For each participant, one high-value set and one low-value set were chosen to be consistently associated with the cue (Go items), whereas all other stimuli appeared without a cue (No-Go items). The cue appeared following a delay of ~750-ms from stimulus onset. To maintain a balanced difficulty level throughout the training phase, the delay was modified according to participants’ performance, as conducted in previous cue-approach studies^11,12,15^.

### Probe

Preference change following training was evaluated in a probe phase. On each probe trial, two items appeared to the right and left of a central fixation cross and participants were asked to select their preferred stimulus. In each pair, both items were of similar initial value (either high-value or low-value), but only one item was a Go item, associated with a cue during training. For each pair, participants had 1500-ms to select their preferred stimulus, followed by a 500-ms choice confirmation and a fixation cross for a jittered duration with an average of 3000-ms (range of 1000–11000-ms, 1000-ms intervals; see Fig. 2c).

In the design used in Experiments 1–4, probe choices of each value category (high and low value) included eight Go items which were compared to eight No-Go items of equal mean rank, for a total of 64 (8 × 8) unique comparisons per value category. In the second design used in Experiments 5–10, probe choices included six Go and six No-Go items for a total of 36 (6 × 6) unique probe comparisons in each category (Supplementary Figure 1). While previous studies with CAT^11–15^ only included 4 × 4 unique comparisons per value category, in Experiments 1–4 of the current work we modified the design of the training and probe parts to include 8 × 8 Go items, in an attempt to increase the number of unique probe choices. Following experiments 1–4, we hypothesized that a value category of 16 items was too wide, as within each value-category, participants were more likely to choose the relatively higher-value items. Therefore, in the later designs (used in Experiments 5–10) the number of Go items in each value-category was reduced to 6, in order to maintain smaller gaps in initial preferences between all items in the same value category.

In addition to these comparisons, as in previous cue-approach experiments^11–15^, ‘sanity check’ trials were also incorporated in the probe phase. In the ‘sanity check’ trials, participants were asked to choose between pairs of items in which one item was of initial high-value and the other of initial low-value (both Go or both No-Go items), in order to reassure the validity of the initial preference evaluation procedure. The probe phase included two runs. On each run, all unique probe pairs were presented in a random order.

In Experiments 5–6, conducted with snack-food items, choices were made for actual food consumption. To ensure incentive-compatible choices, participants were informed in advance that one trial would be selected at random, and that they would receive the item selected on that trial at the end of the experiment.

### Memory

At the end of the experiment, participants performed an Old/New and Go/No-Go recognition tasks. The results of these tasks are not reported here as they are beyond the scope of this paper.

### Follow-up

In Experiments 2 and 5–8, participants returned after a predetermined mean period of either one, two, four or six months (see Table 1). In the follow-up sessions, participants completed only the probe and memory tasks again. For each participant, the follow-up tasks included the same stimuli and probe pairs he or she had previously performed during the original session, presented in a random order. All participants were notified in advance and encouraged to return to the follow-up sessions.

## Results

**Figure 3:**
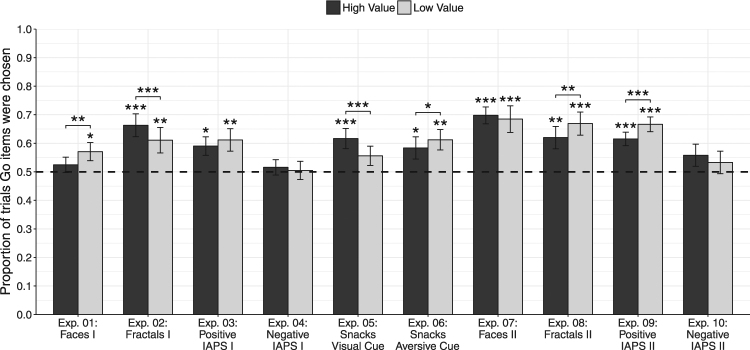
Probe results of Experiments 1–10. Mean proportion of trials participants chose Go items over No-Go items, for high-value (dark gray) and low-value (light gray) probe pairs. Dashed line indicates 50% chance level, error bars represent standard error of the mean. Asterisks reflect statistical significance in a two-tailed logistic regression analysis. Asterisks above each bar represent proportions different from chance (log-odds = 0, odds-ratio = 1). Asterisks above pairs of bars represent differential effect for the two value categories (log-odds = 0, odds-ratio = 1); ***p < 0.001, **p < 0.01, *p < 0.05.

**Figure 4:**
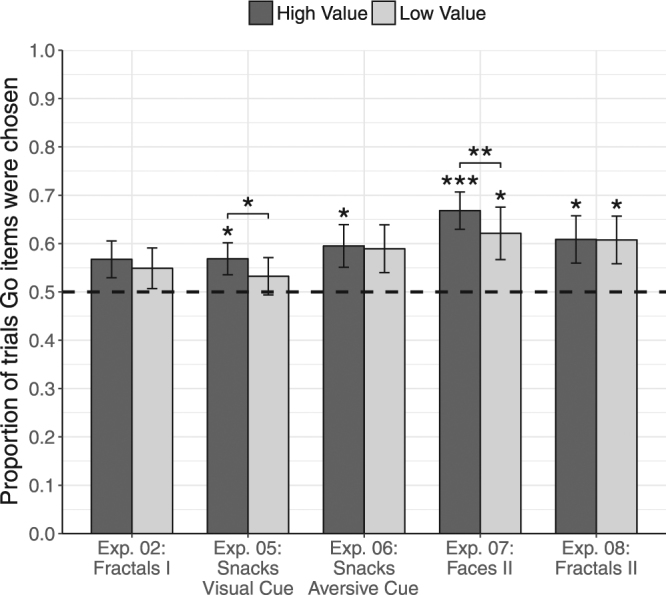
Probe results of follow up sessions. Results from Experiments 2 (115.6 days after training), Experiment 5 (51.8 days), Experiment 6 (183.9 days), Experiment 7 (33.2 days) and Experiment 8 (183.9 days). Mean proportion of trials participants chose Go items over No-Go items, for high-value (dark gray) and low-value (light gray) probe pairs. Dashed line indicates 50% chance level, error bars represent standard error of the mean. Asterisks reflect statistical significance in a two-tailed logistic regression analysis. Asterisks above each bar represent proportions different from chance (log-odds = 0, odds-ratio = 1). Asterisks above pairs of bars represent differential effect for the two value categories (log-odds = 0, odds-ratio = 1); ***p < 0.001, **p < 0.01, *p < 0.05.

To assess preference changes following training, we analyzed the proportion of probe phase trials in which participants preferred the Go items over the No-Go items, using a two-tailed repeated measures logistic regression. In each pair, both items were of similar initial preference based on the baseline evaluation phase. We hypothesized that the cue approach effect would enhance preferences for the Go items above the chance level of 50% of trials (log-odds = 0; odds-ratio = 1). For each logistic regression analysis, the estimated odds ratios (OR) are reported as the corresponding effect sizes along with 95% confidence intervals (CI). Analyses were conducted separately for pairs of high-value items and pairs of low-value items, similar to previous cue-approach studies^11–15^. The results of all experiments are summarized in Table 2, and Figs. 3 and 4. Analyses and visualizations were conducted using lme4^41^ and ggplot2^42^ R packages, and are available online along with the experimental data at osf.io/puxhx.

**Table 2:** Probe Results - Mean Proportion of Trials Participants Chose Go Items Over No-Go Items.

Experiment	Go cue	Training Runs	Mean Proportion Go items were Chosen			
			First session		Follow-up Session	
			High-Value	Low-Value	High-Value	Low-Value
Exp. 1: Faces	Auditory	12	52.4%, *p* = 0.343	57.1%, *p* = 0.023	—	—
Exp. 2: Fractals	Auditory	12	66.3%, *p* = 2.4E^−4^	61.1%, *p* = 0.009	56.7%, *p* = 0.068	54.9%, *p* = 0.208
Exp. 3: Positive IAPS	Auditory	12	59%, *p* = 0.011	61.2%, *p* = 0.007	—	—
Exp. 4: Negative IAPS	Auditory	12	51.6%, *p* = 0.582	50.5%, *p* = 0.877	—	—
Exp. 5: Snacks	Visual	20	61.7%, *p* = 0.001	55.6%, *p* = 0.096	56.9%, *p* = 0.038	53.2%, *p* = 0.317
Exp. 6: Snacks	Auditory Aversive	20	58.4%, *p* = 0.030	61.2%, *p* = 0.002	59.4%, *p* = 0.031	59.0%, *p* = 0.066
Exp. 7: Faces	Auditory	20	69.8%, *p* = 8.8E^−9^	68.5%, *p* = 2.3E^−4^	66.8%, *p* = 4.8E^−5^	62.1%, *p* = 0.027
Exp. 8: Fractals	Auditory	20	62%, *p* = 0.003	66.9%, *p* = 8.5E^−5^	60.8%, *p* = 0.03	60.8%, *p* = 0.032
Exp. 9: Positive IAPS	Visual	20	61.5%, *p* = 1.4E^−6^	66.7%, *p* = 1.2E^−9^	—	—
Exp. 10: Negative IAPS	Visual	20	55.8%, *p* = 0.116	53.3%, *p* = 0.322	—	—

Reported *p* values indicate a significant deviation from chance level (mean proportion = 50%, odds-ratio = 1) in a two-tailed repeated-measures logistic regression analysis.

### Experiments 1–4: Changing Preferences for Non-Consumable Items - Faces, Fractals and Affective Stimuli

The first goal of the current work was to examine CAT’s efficacy in changing preferences for non-consumable stimuli. To achieve this goal, we tested CAT with unfamiliar stimuli of faces in Experiment 1, as well as abstract stimuli of fractal art in Experiment 2. Experiments 3–4 aimed to test if the CAT effect is dependent on the stimuli’s valence by testing the ability to change preferences for positive (Experiment 3) and negative (Experiment 4) affective stimuli.

In Experiment 1, Cue approach training with unfamiliar faces did not result in significant preference for high-value Go items over high-value No-Go items (mean proportion = 52.4%, OR = 1.11, 95% CI [0.89, 1.38], *p* = 0.343); and a small preference of low-value Go items over low-value No-Go items (mean proportion = 57.1%, OR = 1.39, 95% CI [1.05, 1.84], *p* = 0.023; see Fig. 3 and Table 2). A significant difference was found between high and low value probe choices with a greater effect for the low-value items (OR = 0.82, 95% CI [0.73, 0.93], *p* = 0.001).

One of the important differences of this Experiment 1 from previous cue-approach studies^11,12,15^ with appetitive snack-food stimuli was the increase in the range of each value category to 16 items (eight Go and eight No-Go items), in an attempt to increase the number of possible comparisons during the probe phase to obtain better measurements. This increase came with a known cost of introducing choices between pairs of less homogenous initial values. As this experiment was different in design, we additionally performed an analysis to examine the results using only the most extreme stimuli in each category, i.e. the highest four Go and four No-Go stimuli in the high-value category, and the lowest four Go and four No-Go stimuli in the low-value category. These analyses are equivalent to probe-choices in previous studies^11–13^. Examining the extreme choices revealed enhanced preferences for the highest Go items over the highest No-Go items (mean proportion = 57.9%, OR = 1.41, 95% CI [1.04, 1.92], *p* = 0.026), and a similar trend for low-value choices (mean proportion = 57.9%, OR = 1.44, 95% CI [0.97, 2.17], *p* = 0.073).

The results of Experiments 2–4 were also analyzed separately for the highest and lowest probe-choices as has been done in previous studies^11–13^. In these experiments, the additional analyses of the more extreme comparisons showed consistent results with the main analyses for the entire value category. Therefore, for these samples we do not report the additional analysis and they are available online (osf.io/h36vr), along with the data and all other analyses.

In Experiment 2, following CAT with fractal art stimuli, participants consistently preferred the high-value Go items over the high-value No-Go items during probe phase (mean proportion = 66.3%, OR = 2.56, 95% CI [1.55, 4.22], *p* = 2.4E^−4^); similarly, participants consistently preferred the low-value Go items over the low-value No-Go items (mean proportion = 61.1%, OR = 1.92, 95% CI [1.18, 3.14], *p* = 0.009). The effect on preferences was significantly stronger for high-value probe choices over low-value probe choices (OR = 1.31, 95% CI [1.17, 1.46], *p* = 2.2E^−6^).

In Experiment 3, following training with positive IAPS, participants significantly preferred Go over No-Go items, both in the high-value (mean proportion = 59.0%, OR = 1.62, 95% CI [1.12, 2.34], *p* = 0.011) and in the low-value probe choices (mean proportion = 61.2%, OR = 2.06, 95% CI [1.22, 3.46], *p* = 0.007). Differences between high and low value probe choices were trending towards more robust effects for low-value items (OR = −0.1, 95% CI [−0.20, 0.01], *p* = 0.067).

In Experiment 4, conducted with negative IAPS stimuli, participants displayed no preferences for the Go items, neither for the high-value pairs (mean proportion = 51.6%, OR = 1.07, 95% CI [0.85, 1.34], *p = *0.582); nor for the low-value pairs (mean proportion = 50.5%, OR = 1.02, 95% CI [0.77, 1.36], *p* = 0.877). No significant difference between high and low value probe choices was found (OR = 1.05, 95% CI [0.95, 1.16], *p* = 0.316).

### Experiments 5–6: Using Cues of Different Modality and Valence

After examining CAT with different stimuli, we went on to examine whether using an auditory cue is essential in order to induce CAT preference change. Therefore, in Experiment 5, we used a semi-transparent Gabor on top of the snack-food items (See Fig. 1c,d). Following the results of Experiment 4 showing that CAT did not alter preferences for negative stimuli, in Experiment 6 we tested the importance of cue neutrality, i.e. whether CAT could enhance preferences for appetitive snacks, even when cued with an aversive tone. In both Experiments 5 and 6, we used familiar local snack-food items, similar to those used in previous cue-approach studies^11,12,14,15^.

In Experiment 5, following training with a neutral visual cue, participants preferred the high-value Go items over the No-Go items (mean proportion = 61.7%, OR = 1.73, 95% CI [1.25, 2.38], *p* = 8.9E^−4^). A weaker trend of enhanced preferences was found for low-value Go items (mean proportion = 55.6%, OR = 1.30, 95% CI [0.95, 1.77], *p* = 0.096). As in previous studies using snack-food stimuli^11,12,15^, training with a visual cue resulted in a more robust preference modification for high-value snack-food items than for low-value items (OR = 1.29, 95% CI [1.12, 1.49], *p* = 3.2E^−4^).

Using an aversive auditory cue in Experiment 6 resulted in a significant (yet not as strong) enhanced preference for high-value Go items (mean proportion = 58.4%, OR = 1.46, 95% CI [1.04, 2.07], *p* = 0.030) and a significant enhanced preference for low-value Go items (mean proportion = 61.2%, OR = 1.68, 95% CI [1.21, 2.32], *p* = 0.002), with a more robust preference change for low-value items compared with high-value items (OR = 0.85, 95% CI [0.73, 098], *p* = 0.022).

### Experiments 7–10: Reproducibility of Findings

After showing CAT can be used to modify preferences for various non-consumable stimuli, to support the reproducibility of our findings, we aimed to replicate our results in four additional independent samples with improved designs.

In Experiment 7 we performed an additional cue-approach experiment with face stimuli, obtained from a more contemporary dataset^32^, than the stimuli used in Experiment 1. Cue approach training with unfamiliar faces resulted in enhanced preferences for high-value Go items over high-value No-Go items (mean proportion = 69.8%, OR = 2.57, 95% CI [1.86, 3.55], *p* = 8.8E^−9^); as well as low-value Go items over low-value No-Go items (mean proportion = 68.5%, OR = 2.98, 95% CI [1.67, 5.32], *p* = 2.3E^−4^). No differences were found between preferences of Go items between high-value and low-value probe choices (OR = 1.08, 95% CI [0.92, 1.25], *p* = 0.345).

In Experiment 8, training with fractal art images replicated the results of Experiment 2. Participants preferred the high-value Go items over No-Go items (mean proportion = 62.0%, OR = 1.87, 95% CI [1.23, 2.83], *p* = 0.003); as well as the low-value Go items over the low-value No-Go items (mean proportion = 66.9%, OR = 2.52, 95% CI [1.59, 4.00], *p* = 8.5E^−9^). The preference change effect was more prominent in low-value choices compared with high-value (OR = 0.78, 95% CI [0.68, 0.91], *p* = 0.001).

In Experiment 9, the association of positive IAPS stimuli with a neutral visual cue resulted in enhanced preferences for Go items, both in the high-value items (mean proportion = 61.6%, log-odds = 1.64, 95% CI [1.35, 2.02], *p* = 1.4E^−6^) and in the low-value probe choices (mean proportion = 66.7%, log-odds = 2.10, 95% CI [1.65, 2.67], *p* = 1.2E^−9^). Differences between high and low value probe choices were more robust for low-value items (OR = 0.79, 95% CI [0.70, 0.90], *p* = 5.3E^−4^).

In Experiment 10, conducted with negative IAPS stimuli and a visual cue, results did not show enhanced preferences for Go items, neither in the high-value pairs (mean proportion = 55.8%, OR = 1.35, 95% CI [0.93, 1.96], *p* = 0.116), nor in the low-value pairs (mean proportion = 53.3%, OR = 1.20, 95% CI [0.84, 1.71], *p* = 0.322). No significant differences between high and low value probe choices were found (OR = 1.12, 95% CI [0.98, 1.28], *p* = 0.104).

### Long Term Maintenance

In order to evaluate long-term durability of the CAT effect on preferences, all participants from Experiments 2 and 5–8 were encouraged to return for an additional follow-up session, performed one to six months following training (see Table 1 for retention rates). The results of the follow up experiments are listed by the increasing duration of the follow up interval from one to six months.

In a one-month (mean interval 33.2 days) follow-up of Experiment 7 with face stimuli, enhanced preferences for Go items were maintained both in the high-value (mean proportion = 66.8%, OR = 2.23, 95% CI [1.51, 3.27], *p* = 4.8E^−5^) and the low-value probe choices (mean proportion = 62.1%, OR = 2.16, 95% CI [1.09, 4.27], *p* = 0.027; see Fig. 4 and Table 2). The effect was more robust for high-value than low-value probe choices (OR = 1.26, 95% CI [1.06, 1.50], *p* = 0.007).

In Experiment 5 with snacks and the visual cue, results of the follow-up session conducted approximately two months after the initial training session (mean interval 51.8 days), showed that enhanced preferences for the high-value Go items persisted (mean proportion = 56.9%, OR = 1.34, 95% CI [1.02, 1.78], *p* = 0.038), while preferences for low-value Go items remained at chance level (mean proportion = 53.2%, OR = 1.19, 95% CI [0.85, 1.65], *p* = 0.317), as in the immediate probe session. The more robust preference for high-value snack-food items found in the first session, was also sustained in the follow-up session (OR = 1.16, 95% CI [1.00, 1.35], *p* = 0.049).

In Experiment 6 conducted with snack-food stimuli and the aversive auditory cue, following a mean period of two months from training (mean interval 61.5 days), preferences for Go items were maintained in high-value probe choices (mean proportion = 59.4%, OR = 1.52, 95% CI [1.04, 2.24], *p* = 0.031); while in the low-value probe choices, we observed only a trend of enhanced preferences for Go items (mean proportion = 59.0%, OR = 1.53, 95% CI [0.97, 2.42], *p* = 0.066), with no difference between high-value and low-value probe choices (OR = 1.02, 95% CI [0.85, 1.23], *p* = 0.780).

In a four-month (mean interval 115.6 days) follow-up session of Experiment 2 with fractal art stimuli, enhanced preferences were generally maintained for the high-value Go items (mean proportion = 56.7%, OR = 1.36, 95% CI [0.98, 1.89], *p* = 0.068), but not for the low-value Go items (mean proportion = 54.9%, OR = 1.25, 95% CI [0.88, 1.78], *p* = 0.208). Results were not significantly different for high-value versus low-value choices (OR = 0.92, 95% CI [0.81, 1.06], *p* = 0.266).

In a six-month (mean interval 183.9 days) follow-up of Experiment 8 with fractal art stimuli, behavioral change was maintained in both value categories, as participants consistently preferred both the high-value Go items (mean proportion = 60.8%, OR = 1.62, 95% CI [1.05, 2.51], *p* = 0.030), as well as the low-value Go items (mean proportion = 60.8%, OR = 1.62, 95% CI [1.04, 2.50], *p* = 0.032). No difference was found between high and low value probe choices (OR = 1.01, 95% CI [0.85, 1.20], *p* = 0.917).

### Choice Consistency Across Time

In order to examine if the probe choices remained consistent for the same items across time, we tested whether the items chosen in the first session were more likely to be chosen again in the follow-up session. We performed an analysis using a repeated measures logistic regression model, in which each follow-up session probe choice was modelled by the outcome of the same choice in the first session (in which participants could have chosen the Go items either on zero, one or both trials presented in the first probe session). We found that the follow-up choices were consistent across all follow-up sessions (OR > 4.73, *p* < 0.0001 for all experiments, see Table 3).

**Table 3:** Choice Consistency: Regression of Follow-Up Sessions with Choices in the First Session.

Experiment	Mean Proportion Go items were Chosen in Follow-Up Session			OR 95% CI
	Number of times Chosen in First Session			
	0	1	2	
Exp. 2: Fractals	34.8%	54.6%	74.9%	4.73 [3.92, 5.7]
Exp. 5: Snacks	24.0%	47.1%	80.2%	11.87 [9.58, 14.71]
Exp. 6: Snacks	31.6%	57.0%	79.4%	7.84 [6.1, 10.08]
Exp. 7: Faces	27.2%	53.3%	81.8%	8.37 [6.36, 11.02]
Exp. 8: Fractals	27.1%	54.3%	78.2%	8.8 [6.71, 11.55]

Reported OR indicate the logistic regression effect, comparing trials in which the Go items were chosen 0% versus 100% of the first session trials. In all experiments, *p *< 0.0001.

### Effect of Extensive Training

In Experiments 5–10 we performed a more extensive training procedure with 20 training runs, in comparison with the previous Experiments 1–4, in which training included 12 runs. This change in paradigm was done to test if longer training induces a stronger effect. We performed a logistic regression analysis comparing the effects found in Experiments 1–4 with the effects found in Experiments 5–10, using the experiment as a random effect. We did not find a stronger effect in samples using more extensive training, neither in the first sessions of each experiment (OR = 1.24, 95% CI [0.91, 1.68], *p* = 0.165), nor in the follow-up sessions (OR = 1.07, 95% CI [0.74, 1.54], *p* = 0.716).

## Discussion

The current research studies the mechanism and boundary conditions of the novel non-externally reinforced cue-approach task. In 10 independent samples with 255 participants, we showed for the first time that CAT can be used to enhance preferences for non-consumable stimuli including faces, fractal art images and positive affective stimuli. We showed that a visual cue can be used to induce the effect as well as an aversive tone with appetitive snack-food items. As a boundary condition we found, in two independent samples, that preferences towards negative affective stimuli could not be changed. In a series of five follow-up sessions, performed one to six months after training, we found that the single 40-minute training session led to a long-lasting preference change. These findings shed light on the mechanism as well as boundaries of this non-reinforced behavior change paradigm.

In this study, we found that CAT was effective in increasing preferences for unfamiliar faces, fractals and positive emotional IAPS images. In the first faces experiment (Experiment 1) we found an inconclusive trend of preference change for stimuli of lower quality and presumably a too-wide value range; therefore, in an improved replication (Experiment 7) we changed the stimuli and narrowed the value category. This induced a stronger and more consistent preference change. The ability to change preferences for abstract stimuli, such as fractals (Experiments 2 and 8), exemplifies the generalizability of the non-reinforced behavioral change mechanism underlying CAT, which goes beyond stimuli of consumable, familiar and even tangible nature.

Using affective stimuli in Experiments 3–4 and 9–10, we found that cue approach can modify preferences for stimuli of positive valence (Experiments 3 and 9). However, we were not able to induce this behavioral change for negative affective stimuli, neither with an auditory cue nor with a visual cue (Experiments 4 and 10, respectively). Replicating these null results with a visual cue assisted in ensuring that participants were looking at the stimuli during training. This was especially important in experiments with negative affective stimuli, where participants might have been motivated to avert their gaze from the unpleasant stimuli during training, which could provide alternative explanation for the null results. These findings suggest an important boundary condition of CAT, showing that the association of a Go cue can be used to change preferences for positive but not negative affective stimuli. These findings correspond with similar results with operant conditioning, which showed that go responses were better learned by association with rewards, while no-go responses were better learned by association with punishments^19–21^. Future studies could test this hypothesis using a non-reinforced paradigm that would require a no-go response, in order to modify preferences for negative affective stimuli.

Using familiar local snack-food items with a neutral visual cue and an auditory aversive cue (Experiments 5–6), we found that the CAT effect is not limited to a specific cue modality. In Experiment 5, we showed that using a visual cue rather than the original neutral tone led to similar results as in previous cue-approach studies with local snacks^11,12^. These results suggest that modifying the modality of the cue from auditory to visual does not impair the behavioral change effect. Interestingly, an association with an aversive auditory cue (Experiment 6), which might have been expected to decrease preferences for associated items via classical conditioning^25^, resulted in enhanced preferences for both high and low value Go items. Using an aversive cue resulted in a significantly stronger preference modification for the less liked low-value snack-food items. This was surprising given that in other cue-approach experiments conducted with snack-food items there was no change or a weaker change for low-value snack-food items^11,12^. This might suggest an important interaction between stimuli and cue, as a less-positive Go cue may be more effective in modifying preferences for less-favorable stimuli. Future research could directly test the effect of high versus low value stimuli using additional aversive cues, such as unpleasant tactile or electric stimulation. Furthermore, collecting the subjective perception of the unpleasantness of the cue, could shed further light on the interplay between its unpleasantness and behavioral change.

In previous studies conducted with snack-food items^11,12^, preference change was found to have a differential effect as a function of value category: a stronger change of preferences was found for higher-value snacks, and a relatively weaker change was found for lower-value snacks. This effect was not found in experiments that used CAT to change preferences towards fruits and vegetables^13^. In the current work, in samples using non-consumable items (faces, fractals and affective stimuli) we also did not observe such a consistent differential effect. This might suggest that the greater change for higher-value items is linked to previous experience, such as existing preferences for familiar snack-food items. Our results imply that the mechanism underlying CAT may be used to modify preferences for the entire neutral and positive value range of unfamiliar stimuli, for which participants had no previous experience. Our null results with the negative affective stimuli experiments, may resemble the differential effect for high-value versus low-value snack-food items, as in both cases training the less positive stimuli did not induce an enhancement of preferences. These findings hedge the boundaries of the learning mechanism underlying CAT, suggesting it may be specific to neutral and positive rather than negative stimuli, even when using an aversive cue. The consistent findings that the task had a weaker or no effect on preferences for less liked snack-food items in the current and previous studies corresponds with the two experiments using negative affective stimuli. Together, they serve as evidence against a concern that the experimental results may stem from participants trying to affirm the researchers’ aim due to demand characteristics^43^. Taken together, the lack of ability to change preferences for lower value items suggests a potential thresholding mechanism, such that CAT can only affect items above a certain initial preference level^44^. We hypothesize that in the present work we did not obtain a differential CAT effect between high and low value items, as was previously found in experiments with snack-food items^11,12^, due to two potential reasons. First, it is possible that the effect relies on preferences based on prior experiences, as in subjective valuation of snack-food items. This could be directly examined with non-consumable items in future studies by comparing unfamiliar to familiar stimuli (e.g., politicians or movie stars). Second, there might have been greater variance and range of values in those items. This could be tested using a wider value spectrum of non-consumable items within the same experimental sample, e.g. by incorporating in the same experiment both positive and negative affective IAPS stimuli, or faces with a variety of facial emotional-expressions.

We examined the impact of extending training by comparing results of Experiments 1–4 with 12 training runs versus Experiments 5–10, with 20 training runs. Extending the training did not induce a significantly stronger CAT effect. This suggests CAT yields a robust effect already at 12 training runs. Future studies could directly test the effect of training duration by manipulating the duration within the same cue-approach experiment and modelling the effect on subsequent preference change.

In five of the experiments, we included a follow-up session, one to six months after training. In all cases, the behavioral change effect persisted over time. These results point to the high durability of behavioral change induced without external reinforcements or context change. We found that choices across sessions were highly consistent between the first and follow-up sessions, suggesting that the effects are item-specific and remain stable across a long period of time. A relatively short 40-minute training session successfully affected preferences, which then persisted over long periods of time, up to six months after training, with no maintenance procedures between these time points. Thus, this non-reinforced mechanism could be valuable for designing effective real-world interventions that could induce a long-lasting impact. Our results demonstrate that CAT changed long-term preferences for fractal art images, which are unfamiliar visual stimuli for which participants had no prior experience or affective load. This, putatively suggests that the training induces a change in lower-level brain areas^29^ that lingers for several months, e.g. visual processing areas in the occipital and temporal lobes. This hypothesis will need to be tested directly in future functional imaging studies.

The work presented here serves as foundation for studies examining real-world applications of CAT. Future studies should empirically test the generalizability of the findings discussed in the current work to real-life behavior and different contexts, beyond the controlled laboratory environment. Applicable implementation of CAT can be used to enhance desired behaviors over less desired ones, not necessarily limited to food consumption. In the clinical field, several psychological disorders such as depression and anxiety are characterized by a cognitive bias towards negative affective social stimuli^45,46^. Attentional bias modification treatments for these disorders have been tested in an attempt to improve clinical symptoms^47,48^. Similarly, the general mechanism underlying CAT may be used to induce a counter-bias and enhance preferences for positive affective stimuli. Such preference modification may, in turn, lead to congruent changes in positive mood with beneficial long-term therapeutic effects.

In conclusion, in the current work, we show that the cue approach task, previously shown to change preferences for consumable food items, has a wide reach beyond specifics stimuli and cues. We demonstrate that the behavioral change induced by a short 40-minute training procedure is durable over long periods of several months after training. Our findings suggest that non-externally reinforced behavioral change holds great potential to develop novel applications with long term efficacy, which can be used to enhance desired behavior in a wide array of domains.

## Electronic supplementary material

Supplementary Information
