# Supplementary material for: The Cue-Approach Task as a General Mechanism for Long-Term Non-Reinforced Behavioral Change
Source: Sci Rep. 2018 Feb 26;8:3614. doi: 10.1038/s41598-018-21774-3 (PMC5827734; doi:10.1038/s41598-018-21774-3)
Supplement: Supplementary file 1 — Supplementary Information [file 41598_2018_21774_MOESM1_ESM.pdf]

The Cue-Approach Task as a General Mechanism for Long-Term Non-Reinforced Behavioral Change:

Supplementary Information

Tom Salomon[1], Rotem Botvinik-Nezer[1,2], Tony Gutentag[3], Rani Gera[1], Roni Iwanir[1], Maya Tamir[3], Tom Schonberg*[1,2]

[1]Faculty of Life Sciences, Department of Neurobiology, Tel Aviv University. [2]Sagol School of Neuroscience, Tel Aviv University. [3]Department of Psychology, Hebrew University of Jerusalem

*Corresponding author

Supplementary Table 1

Participants Disqualification Criteria

Experiment	Go cue	Sample Size (Excluded)	Number of Disqualified Participants
Exp. 1: Snacks	Auditory	19 (12)	9 - Training [a]<br>3 - Apparatus [b]
Exp. 2: Fractals	Auditory	25 (3)	2 - Apparatus<br>1 - Intransitive ranking
Exp. 3: Positive IAPS	Auditory	27 (0)	
Exp. 4: Negative IAPS	Auditory	28 (5)	1 - Participant requested to stop<br>4 - Training
Exp. 5: Snacks	Visual	25 (1)	1 - Training [a]
Exp. 6: Snacks	Auditory Aversive	25 (4)	3 - Apparatus<br>1 - Avoided probe choices of low-value items
Exp. 7: Faces	Auditory	25 (1)	1 - Training
Exp. 8: Fractals	Auditory	25 (2)	1- Apparatus<br>1- Training
Exp. 9: Positive IAPS	Visual	29 (3)	1 - Participant requested to stop<br>2 - Training
Exp. 10: Negative IAPS	Visual	27 (3)	3 - Training

Note. A total of 35 participants were disqualified from analysis due to reasons mentioned above.
[a] The most common cause of disqualification was behavior during training. Participant that during training phase stopped responding to the Go cue for prolonged periods of time. This exclusion criteria was adopted from previous cue-approach published work [11,12]. In Experiment 1, this criterion was applied post-hoc, which resulted in a relatively smaller number of participants.
[b] Another frequent reason for disqualification was due to technical problems with the apparatus running the experiment.

Supplementary Table 2

IAPS Stimuli Used in Experiments 3-4 and 9-10

	**Experiments 3 and 9: Positive Valence**						**Experiments 4 and 10: Negative Valence**				
	**Code**	**Description**		**Code**	**Description**		**Code**	**Description**		**Code**	**Description**
**1**	1540	Cat	**31**	5982	Sky	**1**	*	Foot	**31**	7359	PieW/bug
**2**	1590	Horse	**32**	7200	Brownie	**2**	*	Spiders	**32**	8480	BikerOnFire
**3**	1660	Gorilla	**33**	7260	Torte	**3**	*	Bugs	**33**	8485	Fire
**4**	1720	Lion	**34**	7291	Chicken	**4**	1019	Snake	**34**	9008	Needle
**5**	1721	Lion	**35**	7390	IceCream	**5**	1111	Snakes	**35**	9042	StickThruLip
**6**	2030	Woman	**36**	7430	Candy	**6**	1271	Roaches	**36**	9050	PlaneCrash
**7**	2058	Baby	**37**	7470	Pancakes	**7**	1274	Roaches	**37**	9120	OilFires
**8**	2155	Pregnant	**38**	7480	Pasta	**8**	1304	AttackDog	**38**	9290	Garbage
**9**	2209	Bride	**39**	7482	Lamb	**9**	2039	Woman	**39**	9291	Garbage
**10**	2345	Children	**40**	7499	Concert	**10**	2205	Hospital	**40**	9300	Dirty
**11**	2347	Children	**41**	7508	FerrisWheel	**11**	2455	SadGirls	**41**	9301	Toilet
**12**	2352.1	Kiss	**42**	8001	Basketball	**12**	2456	CryingFamily	**42**	9321	Vomit
**13**	2362	Girl&Dog	**43**	8090	Gymnast	**13**	2457	CryingBoy	**43**	9322	Vomit
**14**	2398	Boat	**44**	8130	PoleVaulter	**14**	2683	War	**44**	9325	Vomit
**15**	2550	Couple	**45**	8158	Hiker	**15**	2691	Riot	**45**	9340	Garbage
**16**	2655	Child	**46**	8170	Sailboat	**16**	2799	Funeral	**46**	9341	Pollution
**17**	4597	Romance	**47**	8208	Surfer	**17**	3103	Injury	**47**	9342	Pollution
**18**	4610	Romance	**48**	8280	Diver	**18**	3160	EyeDisease	**48**	9373	Garbage
**19**	4612	Couple	**49**	8340	Plane	**19**	6210	AimedGun	**49**	9390	Dishes
**20**	4614	Romance	**50**	8371	Rafting	**20**	6242	Gang	**50**	9395	Dishes
**21**	4619	Romance	**51**	8380	Athletes	**21**	6312	Abduction	**51**	9425	Assault
**22**	4628	Wedding	**52**	8420	Tubing	**22**	6370	Attack	**52**	9427	Assault
**23**	4640	Romance	**53**	8461	HappyTeens	**23**	6560	Attack	**53**	9490	Corpse
**24**	4641	Romance	**54**	8467	Runners	**24**	6562	Attack	**54**	9495	Fire
**25**	5199	Garden	**55**	8470	Gymnast	**25**	6570	Suicide	**55**	9561	SickKitty
**26**	5260	Waterfall	**56**	8499	Rollercoaster	**26**	6571	CarTheft	**56**	9600	Ship
**27**	5480	Fireworks	**57**	8500	Gold	**27**	6821	Gang	**57**	9622	Jet
**28**	5626	HangGlider	**58**	8502	Money	**28**	6836	Police	**58**	9903	CarAccident
**29**	5814	Mountain	**59**	8510	SportCar	**29**	6838	Police	**59**	9909	BurningCar
**30**	5829	Sunset	**60**	8540	Athletes	**30**	7078	Bucket	**60**	9930	ShipWave

Note. In Experiments 4 and 10 using negative affective stimuli, we used 3 additional negative affective stimuli, not from the IAPS dataset. The valence and arousal norms for these stimuli were assessed in another independent work.

Supplementary Table 3

Snack food Stimuli Used in Experiments 5-6

	Brand	Description		Brand	Description
**1**	Apropo	Salty snack	**31**	Halva	Halva
**2**	BabyDoll	Candy	**32**	HappyHippo	Chocolate
**3**	BagaleShtuhim	Salty snack	**33**	HispusimShatiah	Gummy candy
**4**	Bamba	Salty snack	**34**	Hit	Cookies
**5**	BambaNugat	Sweet and salty snack	**35**	KashitSour	Gummy candy
**6**	BambaSweet	Sweet and salty snack	**36**	Keifli	Salty snack
**7**	BisliGrill	Salty snack	**37**	KifkefMaklot	Chocolate
**8**	BisliOnion	Salty snack	**38**	KinderBuenoBrown	Chocolate
**9**	BisliPizza	Salty snack	**39**	KinderBuenoWhite	Chocolate
**10**	Bounty	Chocolate	**40**	KinderJoy	Chocolate
**11**	Cheetos	Salty snack	**41**	Kitkat	Chocolate
**12**	ChocolateParaCookies	Chocolate	**42**	Loacker	Wafer
**13**	ChocolateParaMarir	Chocolate	**43**	Mars	Chocolate
**14**	ChocolateParaMilk	Chocolate	**44**	Mekupelet	Chocolate
**15**	ChocolateParaSucariot	Chocolate	**45**	Mentos	Candy
**16**	ClickBalls	Chocolate	**46**	PesekZman	Chocolate
**17**	ClickBisquit	Chocolate	**47**	Popco	Sweet and salty snack
**18**	ClickBlackWhite	Chocolate	**48**	Shugi	Energy bar
**19**	ClickXLbrown	Chocolate	**49**	SkittlesFruits	Candy
**20**	ClickXLwhite	Chocolate	**50**	SkittlesSour	Candy
**21**	CrunchBisquitBrown	Chocolate	**51**	Smarties	Candy
**22**	CrunchBisquitWhite	Chocolate	**52**	Snickers	Chocolate
**23**	CrunchShokoVanil	Chocolate	**53**	Taami	Chocolate
**24**	DoritosGrill	Salty snack	**54**	Tapuchips	Salty snack
**25**	DoritosNatural	Salty snack	**55**	TapuchipsCrunch	Salty snack
**26**	DoritosSourSpicy	Salty snack	**56**	Tictac	Candy
**27**	Dubonim	Salty snack	**57**	Tortit	Chocolate
**28**	Egozi	Chocolate	**58**	Twist	Wafer
**29**	GumiSnakes	Gummy candy	**59**	Twix	Wafer
**30**	GumiWine	Gummy candy	**60**	WerthersOriginal	Candy

Note. The complete images data set is available online:
http://schonberglab.tau.ac.il/resources/snack-food-image-database/

a. Sorted Ranking	Item		b. pairs: High Go		High No-Go
.98	1		7		8
.95	.		10		9
.89	.		12		11
.	7	High-value Items 7:22	13	X	14
.	.		15		16
.	.		18		17
.	.		20		19
.	22		21		22
.	.				
.	.		Low Go		Low No-Go
.	39	Low-value Items 39:54	39		40
.	.		42		41
.	.		44		43
.	.		45	X	46
.	54		47		48
.12	.		50		49
.09	.		52		51
.03	60		53		54

c. Sorted Ranking	Item		d. pairs: High Go		High No-Go
.98	1		7		8
.95	.		10		9
.89	.		12	X	11
.	7	High-value Items 7:18	13		14
.	.		15		16
.	.		18		17
.	18				
.	.		Low Go		Low No-Go
.	.		44		43
.	.		45		46
.	.		47	X	48
.	43	Low-value Items 43:54	50		49
.	.		52		51
.	.		53		54
.	54				
.12	.				
.09	.				
.03	60				

Supplementary Figure 1. Sorting and pair matching procedure.

1a. In the first experimental design (used in Experiments 1-4), items were rank ordered based on initial preferences evaluation procedure, and classified as high-value (ranks 7:22) and low-value items (39:54). 1b. Eight high and eight low-value items were associated with the cue and response during training (assigned to be Go items). In the probe phase, all eight Go items were paired with similar-value No-Go items, forming 64 (8×8) unique pairs in each value category. 1c. In a second experimental design (used in Experiments 5-10) similar rank ordering was conducted. Items were classified as high-value (7:18) and low-value items (43:54). 1d. Six high-value and six low-value items assigned to be Go items during training. In the probe phase, all six Go items were paired with similar initial value No-Go items, forming 36 (6×6) unique pairs in each value category. Condition assignments for Go and No-Go items were counterbalanced across participants in both experimental designs.

```matlab
%% Neutral auditory cue
% features:
wave = sin(1:0.25:1000);
freq = 22254;

% Play the sound
sound(wave,freq);

%% Aversive auditory cue
% features:
wave = cot(1:0.25:7541);
freq = 100544;

% Play the sound
sound(wave,freq);
```

Supplementary Code. Neutral and aversive auditory cues, implemented in MATLAB.

In Experiments 1-4 and 7-8 a neutral auditory cue of 180-ms was produced using a sinus wave function. To induce aversive auditory cue in Experiment 6, a longer duration of 300-ms cotangent wave function was used.
